# The Nomogram of Clitoral Length and Width in Iranian Term and Preterm Neonates

**DOI:** 10.3389/fendo.2020.00297

**Published:** 2020-06-03

**Authors:** Mohammadreza Alaei, Farzaneh Rohani, Elahe Norouzi, Nahid Hematian Boroujeni, Roya Isa Tafreshi, Hamid Salehiniya, Fahimeh Soheilipour

**Affiliations:** ^1^Department of Pediatric Endocrinology and Metabolism, School of Medicine, Shahid Beheshti University of Medical Sciences, Tehran, Iran; ^2^Pediatric Growth and Development Research Center, Institute of Endocrinology and Metabolism, Iran University of Medical Science, Tehran, Iran; ^3^Department of Pediatrics, Iran University of Medical Sciences, Tehran, Iran; ^4^Department of Pediatrics, School of Medicine, Iran University of Medical Sciences, Tehran, Iran; ^5^Social Determinants of Health Research Center, Birjand University of Medical Sciences, Birjand, Iran; ^6^Minimally Invasive Surgery Research Center, Iran University of Medical Sciences, Tehran, Iran

**Keywords:** newborn, preterm, term, clitoris, clitoromegaly, Iran

## Abstract

**Background and Objectives:** Clitoromegaly is an important parameter in the evaluation of ambiguous genitalia in neonates, but the normative data for clitoral size in newborns have racial/ethnic differences. The present study aimed to determine clitoral length (CL) and clitoral width (CW) values and establish cutoff measurement to define clitoromegaly in both term and preterm Iranian neonates for the first time.

**Methods:** A total number of 580 female newborn infants delivered at 28–42 weeks of gestation were enrolled in the study, and their CL and CW were measured on the first 72 h of birth. Data about birth weight (BW), body length (BL), and head circumference (HC) of newborns; mothers' age; and gestational age (GA) were recorded, too. Results were presented as mean ± standard deviation (SD) for quantitative variables and were summarized by frequency (percentage) for categorical variables. Backward stepwise regression analysis was used for prediction of CL and CW.

**Results:** Among 580 Iranian female newborns studied, 187 were term neonates and the other 393 newborns were preterm. Mean ± SD values of CL were 6.11 ± 0.39 mm in term infants and 5.45 ± 0.64 mm in preterm infants (*P* < 0.001). Mean ± SD values of CW were 4.22 ± 0.43 in term infants and 3.68 ± 0.53 in preterm infants (*P* < 0.001). Regression analysis showed that CL was correlated with GA considered by last menstrual period, BL, BW, and HC; and CW was associated with GA, BL, and BW.

**Conclusion:** This study suggests normative values (mean + 1, 2, and 3 SD) of CL and CW according to GA, which can be used as a reference for Middle East's newborns, especially Iranian newborn babies.

## Introduction

Assessment of external genitalia by performing a careful physical examination in both female and male infant newborns is crucial to diagnose ambiguous genitalia. Clinical findings in an apparent female newborn infant that raise the possibility of disorder of sex development (DSD) include clitoral hypertrophy of any degree or clitoromegaly which defied clitoral length (CL) or clitoral width (CW) above mean + 2 SD, foreshortened vulva with single opening, and inguinal hernia containing a gonad. All of these findings in apparent female infant must raise suspicion to ambiguous genitalia. The differentiation of a 46XX baby with abnormally apparent external genitalia includes congenital adrenal hyperplasia (CAH), as the most common cause, and maternal source of virilization such as drugs with an androgenic effect, hyperthecosis, and placental aromatase deficiency (1–3). On the other hand, apparently prominent clitoris in newborns, especially in premature infants, may lead to overdiagnosis.

As failure to identify and treat CAH may lead to life-threatening and potentially fatal adrenal crisis, well-timed laboratory studies, and emergent medical intervention are of vital importance. Unfortunately, newborn screening for CAH is not available in most developing countries such as Iran. Therefore, accurate evaluation of external genitalia of female infant newborns, particularly clitoral size, is necessary (4, 5). Clitoromegaly is best assessed by measurement of the dimensions of paired corpora cavernosa. CW and CL above 6 and 10 mm, respectively, have traditionally been defined as clitoromegaly (1).

However, ethnic and racial differences in male and female anthropometric external genitalia sizes have been reported (6–11), and existing data may not be applicable to Middle East population especially Iranian newborns, for which no published study exists. As far as the authors are concerned, the present study is the first to investigate the clitoral size in newborn females in Iran, and very few similar studies in Middle East has been undertaken. Normative clitoral size data for healthy term newborns have been reported sparsely from different Asian and Caucasian infants (12–16). Also, only few studies are available, conducted on both term and preterm infants (12, 16). Thus, this study was performed to establish mean and standard deviation (SD) values of CL and CW of Iranian term and preterm newborns in relation to gestational age (GA) and anthropometric measurements.

## Materials and Methods

### Study Design

This cross-sectional study provides normative data on CL and CW in term and preterm neonates from Iran. The parents of 628 children consented to the study, but finally, 580 female newborns satisfied the inclusion criteria and were included.

Inclusion criteria consisted of live female neonates under 72 h of age, with GA of 28–42 weeks and without major congenital malformations/dimorphism and apparent genital anomalies. Neonates who had >2 weeks' discrepancy in calculation of GA by first-trimester ultrasound or last menstrual period by the Ballard scoring system were excluded. Neonates who met the inclusion/exclusion criteria were included into the study by the convenience sampling method until saturation of the sample size.

Data collected included infants' CL and CW, birth body weight (BW), body length (BL), head circumference (HC), GA considered by last menstrual period, type of delivery, mother's age (MA), singleton/twin/multiple pregnancies, and drug and medical history (gestational diabetes and preeclampsia) of mother during pregnancy.

Clitoral size was measured in a 37°C environmental temperature and sufficient light, while the newborns were put in supine position with both hips flexed and perineum adequately exposed. Then, the labia majora were separated, and the prepuce of the clitoris was gently retracted. Clitoral size measurement was performed as described by Verkauf et al. (17). CW was measured in the greatest transverse diameter of the clitoris by a digital caliper (Aesculap, Center Valley, PA, USA) with a resolution of 0.01 mm twice for every infant, and the mean was recorded. Neonate's BL was measured by an infantometer (Harpenden, London, UK), with 1-cm resolution (18), and neonate's BW was measured by a digital weighing scale (Seca, Hamburg, Germany) to the nearest 10 g. GA was calculated by first-trimester ultrasound or last menstrual period verified if necessary by the Ballard scoring system (19).

### Ethical Considerations

The protocol of the study was approved by the Ethics Committee of Iran University of Medical Sciences. The design and objectives of the study were explained to the parents of all participants, and written informed consent was obtained from those who were willing to have their neonates participate in the study, and it was clarified that their data would be kept confidential and analyzed anonymously.

### Statistical Analysis

Results were presented as mean ± SD for quantitative variables and were summarized by frequency (percentage) for categorical variables. The correlation of variables was tested by correlation analysis, and backward regression analysis was used for prediction of CL and CW. For the statistical analysis, the statistical software SPSS version 16.0 for Windows (SPSS Inc., Chicago, IL, USA) was used. *P*-values of 0.05 or less were considered statistically significant.

## Results

In this study, 580 female newborns were enrolled. Among a total of 580 neonates, 187 (32.2%) neonates were born term (≥38 weeks) and 393 (67.8%) were born preterm (<38 weeks). Mean MA of the included neonates was 25.18 ± 0.45. The mean ± SD value of neonates' BW was 2,709 ± 788 g. Mean ± SD values of BL and HC were 46.9 ± 3.99 and 32.49 ± 3.26 cm, respectively. Among all participants, 32.75% (*N* = 190) were born by cesarean section and 67.2% (*N* = 390) by vaginal delivery. A total of 87.1% were singleton pregnancies, 9.3% were twin pregnancies, and 3.4% were triplets.

Mean ± SD values of CL were 6.11 ± 0.39 mm in term infants and 5.45 ± 0.64 mm in preterm infants (*P* < 0.001). Mean ± SD values of CW were 4.22 ± 0.43 in term infants and 3.68 ± 0.53 in preterm infants (*P* < 0.001). Details of ±1, ±2, and ±3 SD CL and CW are demonstrated in Table 1 based on different GA of neonates.

**Table 1:** Mean and SD of clitoral width and length based on different gestational ages of neonates.

**Gestational age (week)**		**Number**	**Mean**	**Mean + 1 SD**	**Mean + 2 SD**	**Mean + 3 SD**
28–30	Width	30	3.28	3.87	4.46	5.05
	Length		5.03	5.91	6.79	7.67
30–32	Width	57	3.32	3.68	4.04	4.4
	Length		5.03	5.52	6.01	6.5
32–34	Width	74	3.41	3.77	4.13	4.49
	Length		5.14	5.65	6.16	6.67
34–36	Width	104	3.69	4.17	4.65	5.13
	Length		5.53	6.12	6.71	7.3
36–38	Width	128	4.08	4.5	4.92	5.34
	Length		5.83	6.29	6.75	7.21
>38	Width	187	4.21	4.64	5.07	5.5
	Length		6.11	6.49	6.87	7.25

Regression analysis showed that CL was correlated with MA, GA, and neonate's BW, BL, and HC. It also showed that CW was correlated with GA, BL, and BW of newborns. Authors found that CW and CL changes were due mostly to BW (*P* = 0.001). In other words, in preterm newborns, clitoral size is more related to body size rather than to GA.

According to the significant correlation of CW with GA; infant BW, BL, and HC; and MA at delivery, specific regression equations for prediction of clitoral size were generated as follows:

Expected CW (mm) = 0.17 + 0.025 GA (week) + 0.05 BL (cm) + 0.16 BW (kg).

Expected CL (mm) = 2.08 – 0.008 MA (year) + 0.25 GA (week) + 0.028 BL (cm) + 0.027 HC (cm) + 0.26 BW (kg).

## Discussion

The present study determined mean and SD values of CL and CW in a large number of term and preterm Iranian neonates and its correlation with different variables including MA and GA and anthropometric indices.

**Figure 1:**
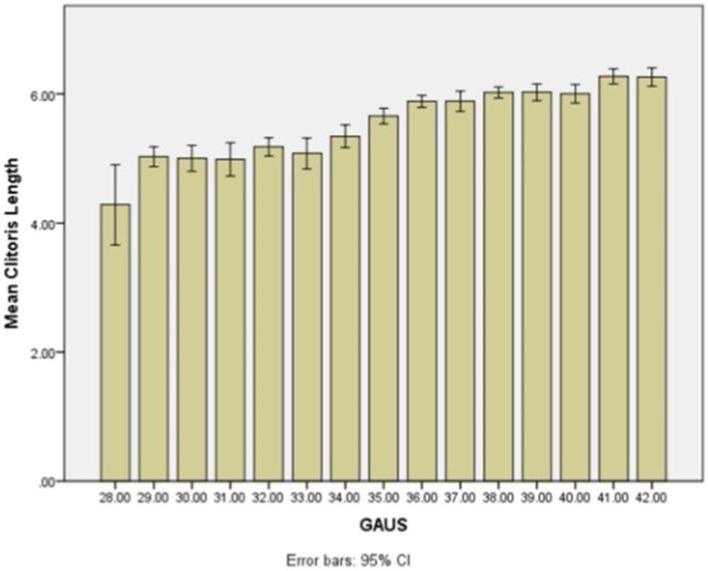
Mean and SD of clitoral length based on different gestational ages of neonates.

**Figure 2:**
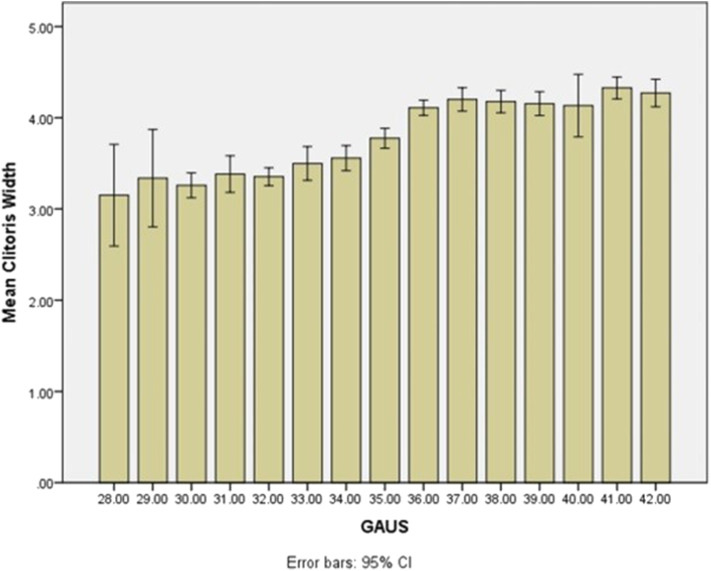
Mean and SD of clitoral width based on different gestational ages of neonates.

A neonate has to have either CW or CL above mean + 2 SD for GA values to be defined as clitoromegaly. Mean + 2 SD values of CW and CL were 5.07 and 6.87 mm, respectively, in term newborn infants with GA of 38 weeks or greater. Authors defined CW and CL of >5 or 7 mm as cutoff values suggesting clitoromegaly in Iranian term newborns. As the authors found, this is the first description of the length and width of neonatal clitoris in Iran, and also one of the largest cohort of both preterm and term clitoral sizes in published literature. Although this study was performed in referral academic hospitals with a large number of preterm births and a high percentage of preterm newborns enrolled, nevertheless, the term neonate sample size is comparable with studies performed previously. Figures 1, 2 demonstrate mean CL and CW by GA in the neonatal population studied. A summarized review of previous similar studies from various ethnicities and population performed on CL, CW, or both in term/preterm female newborns with similar measurement techniques is listed in Table 2. The differences in CL and CW results among different studies may be related to ethnicity but also to different technical methods used for measuring clitoral size (12–16). Furthermore, studies do not make the distinction between term and preterm newborns, as was done in this study, which can explain some of the differences in results.

**Table 2:** Studies investigating clitoromegaly.

**Authors**	**Country**	**Year**	**Term/preterm**	**No**.	**Evaluated parameter**	**Cutoff (mm)**	**Mean + 2 SD (mm)**
Riley and Rosenbloon (20)	USA	1980	Term and preterm	?	CL	_	3.66 + 0.26
Oberfield et al. (21)	USA	1989	Term	82	CL and CW	10 and 6	4 + 2.48 and 3.32 + 1.56
Litwin et al. (16)	Israel	1990	Term and preterm	287	CL	_	_
Jarrett et al. (15)	Nigeria	2010	Term	251	CL and CW	_	7.5 + 3.6 and 4.4 + 1.78
Kutlu et al. (14)	Turkey	2011	Term	325	CL	8	4.93 + 3.22
Mondal et al. (12)	India	2016	Term and preterm	378	CL	6	_
Asafo-Agyei et al. (13)	Ghana	2017	Term	612	CL and CW	_	4.13 + 3.2 and 4.21 + 2.2

*CL, clitoral length; CW, clitoral width*.

Clitoromegaly has not been defined for different GAs and ethnicities in the text or literature definitely. Studies conducted in Asian countries reported various results. A Turkish study performed on 325 term female neonates reported the CL mean ± SD of Turkish newborns as 4.93 ± 1.61 mm. They found that cases with CL of over 8 mm at 97th percentile must be monitored (14).

In another Asian study conducted on 378 female term and preterm newborns in India, the mean ± SD of CL was found to be 3.1 ± 1.54 mm, and a CL cutoff of 6 mm as a criterion for clitoromegaly was suggested (12). None of these two Asian studies assessed CW as a criterion of clitoromegaly.

In the largest cohort of newborn clitoral sizes in published literature, conducted on 612 term female infants in Ghana, the mean ± SD values of CL and CW were 4.13 ± 1.6 and 4.21 ± 1.1 mm, respectively (13). In another study performed in Nigeria, in 251 indigenous term newborns, the mean ± SD of CL was 7.5 ± 1.8 mm, while the mean ± SD of CW was 4.4 ± 0.89 mm (15).

Among studies performed in the United States, the mean ± SD values of term CL were 4 ± 1.24 mm reported by Oberfield et al. (21) and 3.66 ± 0.13 mm reported by Riley and Rosenbloom in Black term and preterm female newborns (20).

As mentioned above, the CL and CW cutoff for clitoromegaly significantly varied among different ethnic populations. So it is essential for neonatologists, health staff, and researchers of each country to refer to the values relevant for their country/race.

In conclusion, the results of the present study on a large number of both term and preterm Iranian female neonates suggested the first nomograms (±1, 2, and 3 SD) for CL and CW by GA, which can be used as a reference for Middle East, especially Iranian, female newborns.

## Data Availability Statement

The datasets generated for this study are available on request to the corresponding author.

## Ethics Statement

The studies involving human participants were reviewed and approved by Ethics committee of Iran University of Medical Sciences, Tehran, Iran. Written informed consent to participate in this study was provided by the participants' legal guardian/next of kin.

## Author Contributions

All authors have approved the final article. The conception and design of the study, or acquisition of data, or analysis and interpretation of data: MA, FR, EN, NH, RT, HS, and FS. Drafting the article or revising it critically for important intellectual content: MA, FR, EN, NH, RT, HS, and FS.

## Conflict of Interest

The authors declare that the research was conducted in the absence of any commercial or financial relationships that could be construed as a potential conflict of interest.
